# Therapeutic Properties of Ayahuasca Components in Ischemia/Reperfusion Injury of the Eye

**DOI:** 10.3390/biomedicines10050997

**Published:** 2022-04-26

**Authors:** Anna Szilágyi, Barbara Takács, Réka Szekeres, Vera Tarjányi, Mariann Bombicz, Dániel Priksz, Attila Kovács, Béla Juhász, Ede Frecska, Zoltán Szilvássy, Balázs Varga

**Affiliations:** 1 Department of Pharmacology and Pharmacotherapy, University of Debrecen, Nagyerdei Krt 98, H-4032 Debrecen, Hungary; dr.szilagyi.anna@med.unideb.hu (A.S.); takacs.barbara@pharm.unideb.hu (B.T.); szekeres.reka@med.unideb.hu (R.S.); tarjanyi.vera@med.unideb.hu (V.T.); bombicz.mariann@pharm.unideb.hu (M.B.); priksz.daniel@pharm.unideb.hu (D.P.); juhasz.bela@med.unideb.hu (B.J.); szilvassy.zoltan@med.unideb.hu (Z.S.); 2 Department of Psychiatry, Faculty of Medicine, University of Debrecen, Nagyerdei Krt 98, H-4032 Debrecen, Hungary; kovacsa@med.unideb.hu (A.K.); frecska.ede@med.unideb.hu (E.F.)

**Keywords:** DMT, harmaline, MAO-A inhibition, retinal ischemia–reperfusion injury, ERG, PARP1, NFκB, HSP70, GFAP, MMP9

## Abstract

Ischemic eye diseases are major causes of vision impairment. Thus, potential retinoprotective effects of N’N-dimethyltryptamine (DMT) were investigated. To inhibit its rapid breakdown by monoamine-oxidase A (MAO-A) enzyme, DMT was co-administered with harmaline, a β-carboline in the Amazonian Ayahuasca brew. Using ligation, 60 min of ischemia was provoked in eyes of rats, followed by 7 days of reperfusion whilst animals received harmaline alone, DMT + harmaline, or vehicle treatment. After 1 week of reperfusion, electroretinographical (ERG) measurements, histological analysis, and Western blot were performed. Harmaline alone exhibited retinoprotection in ischemia–reperfusion (I/R) which was, surprisingly, counterbalanced by DMT in case of co-administration. As both MAO-A inhibition and DMT increase serotoninergic tone synergistically, communicated to be anti-ischemic, thus, involvement of other pathways was investigated. Based on our experiments, DMT and harmaline exert opposite effects on important ocular proteins such as PARP1, NFκB, MMP9, or HSP70, each having a critical role in a different mechanism of eye-ischemia-related pathologies, e.g., cell death, inflammation, tissue destruction, and oxidative stress. Since DMT is proclaimed to be a promising drug candidate, its potentially undesirable effect on eye-ischemia should be further investigated. Meanwhile, this experiment revealed the potential therapeutic effect of MAO-A inhibitor harmaline in I/R-related eye diseases.

## 1. Introduction

Retinal ischemia–reperfusion (I/R) injuries occur in several pathological conditions of the retina, such as diabetic retinopathy, severe glaucoma attack, ischemic optic neuropathy, hypertensive retinopathy, and retinal arterial occlusion. The accumulation of reactive oxygen species (ROS), dysregulated neuroinflammation, and retinal ganglion cell (RGC) apoptosis are characteristic of I/R injury leading to irreversible vision impairment [1,2]. Even though reperfusion is crucial for cell survival, it is prone to cause severe damage by oxidative stress and the following abnormal immune response [3]. Effective treatment against I/R-related eye diseases is still unavailable in clinical practice; therefore, it is essential to find a therapeutical agent for preserving retinal function [2]. Ayahuasca is a traditional South-American decoction used in religious rituals for centuries because of its hallucinogenic and assumed therapeutic effects. This psychoactive beverage consists of β-carboline-rich herbs (e.g., Banisteriopsis caapi) traditionally combined with N’N-dimethyltryptamine (DMT)-containing plants (e.g., Psychotria viridis, Diplopterys cabrerana) [4,5]. MAO-A enzyme is expressed in high number in the gastrointestinal tract and central nervous system (CNS), resulting in rapid degradation of DMT by oxidative deamination; however, the vine of B. caapi is rich in MAO-A inhibitor β-carbolines (e.g., harmine, harmaline). As a result, the co-administration of DMT and harmala alkaloids can elongate the half-life of DMT [6].

In the last decade, DMT became a promising candidate for preventing or treating several pathological conditions due to its potent 5-HT_1A_ receptor agonist properties, which give anxiolytic effects and can decrease impulsive behavior whilst facilitating social interactions. These findings supported the decision to apply DMT or DMT-containing Ayahuasca brew to treat post-traumatic stress disorder (PTSD) and depression. Its psychedelic effect is due to its 5-HT_2A_ receptor-activating capacity [7]. In addition, DMT shows Sigma-1 (Sig-1R) receptor agonist activity, which helps cellular (neuronal) survival against oxidative stress whilst also regulating immune responses [8,9]. Sig-1R is also known to control morphogenesis of neurons, such as neurite outgrowth, synaptogenesis, and myelination, so neuroprotective and retinoprotective effects from DMT are expected [7], and it is a promising candidate for the treatment of different forms of I/R injury [10,11].

Harmaline is a β-carboline that functions as a reversible inhibitor of the A-type of the monoamine oxidase (MAO-A) enzyme. This Harmala alkaloid is one of the cardinal components of the vine of B. caapi vine, besides Harmine and tetrahydro-harmine. β-carbolines can potentiate the effect of tryptamines, although their effect is more complex because they may be neurologically active too. Harmala alkaloids demonstrate significant psychoactive properties as well. Due to their MAO-A-inhibitory and CNS-stimulatory effects, the therapeutic properties of Ayahuasca are partly attributable to these alkaloids [7]. Harmaline gained attention in the last decade being able to exert multiple pharmacological effects, including antimicrobial, antiplatelet, antitumoral, hypothermic, and vasorelaxant properties [12]. Recent studies revealed that MAO enzymes have an important role in oxidative stress since, through their oxidative processes, H_2_O_2_, aldehydes, and ROS are formed. Aldehyde intermediates may exert deteriorative effects on biological systems and decrease the aldehyde dehydrogenase activity, further contributing to oxidative stress [13,14].

Our main aim was to test the biological effects of the main components of Ayahuasca on I/R injury of the eye. In the scientific literature, there are no articles about the effect of DMT and harmaline on protein expression, and for this reason we aimed to perform an extensive protein exploration concentrating on proteins which are crucial in maintaining cellular homeostasis.

## 2. Materials and Methods

### 2.1. Animals and Groups

Male 12-week-old Sprague–Dawley (SD) rats, weighing 350–400 g, were purchased for the experiment from Charles River Laboratories International, Inc. (Wilmington, MA, USA). The animals were maintained in a 12 h light/dark cycle at a constant temperature of 24 °C. Animals had free access to water and were fed standard rodent chow (Ssniff Standard rodent chow) ad libitum. Animals received humane care according to the ARVO (Association for Research in Vision and Ophthalmology) Statement for the Use of Animals in Ophthalmic and Vision Research and NIH guidelines. The Institutional Animal Care Committee of the University of Debrecen (No. 12/2019/DEMÁB; 6 August 2019) approved all methods used during the study. After two weeks of acclimatization, SD rats were randomly divided into the following groups (*n* = 10 in each): vehicle-treated (VT), DMT (Lipomed, DMT-943-FB-1000, Lot: 943.1B0.2) and harmaline (Sigma–Aldrich, 51330-1G, Lot#BCCC2186)-treated (DHT), and harmaline (HT)-treated groups (see Table 1). We delivered the DMT solution by osmotic mini pump (ALZET Osmotic Pump, Model 2ML1), which can continuously deliver solutions subcutaneously at a constant rate for 1 week. Our main aim was to maintain a constant DMT concentration in the central nervous system, especially in the eye bulb, and to prevent large fluctuations in drug concentration: we intended to avoid strong hallucinogenic and serotonergic side effects of DMT. The applied daily dose of DMT was 10 mg/kg/day [15,16]. Due to the low solubility of DMT and the limited internal volume of even the largest-available osmotic minipump (2 mL), technically we could not dissolve harmaline as well in the same solution as DMT to fill them together into the minipump. Furthermore, we did not want to risk any possible unknown chemical interaction between DMT and harmaline. Considering the relatively short half-life of harmaline and its reversible inhibitory effect on the MAO-A enzyme, we decided to apply this agent in both treated groups not only one-time but twice a day by oral gavage using 5 mg/kg (10 mg/kg/day) [17]. Harmaline was dispersed in hydroxyethylcellulose mucilage; for this reason, we also applied the mucilage alone in the vehicle-treated group. As the healthy control (HC) group, we used the right (non-ligated) eyes of the vehicle-treated Sprague–Dawley rats, with which we could reduce the number of the sacrificed animals (according to 3R rule of animal ethics).

### 2.2. Ischemia Model

The animals were injected with ketamine/xylazine combination (100/10 mg/kg). After the onset of deep anesthesia (no reaction for pain stimuli), we applied oxibuprocaine-containing topical ocular anesthetic into the left eyeball (Humacain 4 mg/mL eyedrops, Teva Ltd., Debrecen, Hungary). Subsequently, we experimentally provoked ischemic attack on the left eyes of the SD rats with ligation, using the protocol formerly described [18]. In brief, the eye bulbs were slightly pulled out from the orbit, followed by the placement of thick, atraumatic ligature (a polyethylene cannula helped to form a slip knot from a polyester fiber–Mersilene; 2 mm) suture behind the eye bulb. The slip knot surrounded the optic nerve, the retinal blood vessels, and the retrobulbar connective tissue. Ischemia was initiated by tightening the knot, applying pressure on the retinal vessels. Restriction of blood supply was verified macroscopically with ophthalmoscopy. The discoloration of the fundus indicated the onset of global ischemia. The eyes of the animals were protected from desiccation during anesthesia with carbomer-based eye gel (Vidisic, Bausch & Lomb, Berlin, Germany). Ischemia was maintained for 60 min. At the end of the ischemic period, the occluder was released and removed to allow blood flow through the retinal arteries.

### 2.3. Ocular Echography

Retinal blood flow was determined by using the Vevo 3100 ultrasound system (Fujifilm Visualsonics Inc., Toronto, ON, Canada) equipped with an MX550D transducer at 32 MHz frequency. Rats were anesthetized before the procedure, and were positioned (in prone position) on a heated pad at 39 °C temperature. Water-based, inert ultrasound gel (Aquasonic 100, Parkerlab Inc., Fairfield, NJ, USA) was applied on the corneal surface, and the eye was visualized in the longitudinal macula (LMAC) view. Measurements were made when the cornea, iris, and vitreous body were visualized at the maximal volume. Standard Color Doppler traces were recorded at 9 mm depth, and pulsed wave (PW) Doppler images were recorded to evaluate the blood flow in the short posterior ciliary artery (55°, gate size: 0.27 mm). Blood flow was evaluated before (baseline) and after the 1 h I/R injury (reperfusion). Images were analyzed in a semi-quantitative manner (VevoLAB ver. 5.1., Fujifilm Visualsonics Inc., Toronto, ON, Canada), to evaluate the presence of the flow at baseline conditions and at the reperfusion phase.

### 2.4. Osmotic Mini-Pump Implantation

The osmotic mini-pumps were inserted into the interscapular region of the animals instantly after the ischemic period because they were still under deep anesthesia. The backs of the animals were shaved on the interscapular region, and the skin was disinfected with 10% povidone–iodine solution (Betadine, Egis Pharmaceuticals PLC, Budapest, Hungary). First, a 1-centimeter-long mid-scapular incision was made; then, we inserted a hemostat into the subcutis tissue. By opening and closing the jaws of the hemostat, we created a pocket for the pump. When the size of the pocket was sufficient, we inserted the mini-pump. We closed the wound with a non-absorbable suture and disinfected the surface again. We performed this procedure on both harmaline-treated and vehicle-treated rats without insertion of mini-pump to guarantee that the animals in the different treatment groups were exposed to the same stressful situations (sham-operation).

### 2.5. Electroretinography

After seven days of reperfusion, electroretinographic measurement (ERG) was performed on 10 rats in each group according to a formerly used method [19]. Animals were anesthetized with a mixture of ketamine/xylazine (100/10 mg/kg). At the onset of deep anesthesia, we provoked mydriasis using cyclopentolate-containing eyedrop (Humapent, Teva Ltd., Debrecen, Hungary). After 20 min of dark adaptation, registration of scotopic electroretinogram was carried out with needle reference electrodes inserted in each earlobe, lens measuring electrodes laid tightly on the cornea using conductive gel, and an earthing electrode inserted into the skin in the centerline of the animal (glabella). On the basis of International Society for Clinical Electrophysiology of Vision (ISCEV) guidelines, ERG was carried out with a stroboscope (10,000 mcd/s/m^2^ or 20 cd/m^2^, 0.5 Hz) illuminating the eyes in darkness [20]. Light flashes generated retinal electrical signals which were led to an amplifier, an analogue-to-digital converter (Bridge Amp and PowerLab, ADInstruments, Sydney, Australia), and then to a computer. Electroretinograms were analyzed with PowerLab Chart software (Version 5.2.2, ADInstruments, Sydney, Australia).

### 2.6. Western Blot

Immediately after sacrificing the animals, their eyes were removed from the orbit and put in liquid nitrogen until further molecular biological examinations (*n* = 4 per group). Whole-eye samples were ground to dust and mixed with homogenization buffer (25 mM Tris, 25 mM NaCl, 1 mM Na-Orthovanadate, 10 mM NaF, 10 mM Na-Pyrophoshate, 10 mM Okadaic acid, 0.5 mM EDTA, 1 mM PMSF protease inhibitor cocktail, and distilled water all from Sigma-Aldrich-Merck KGaA, Darmstadt, Germany), then the solution was further homogenized by a disperser (IKA-WERKE, Staufen, Germany). After centrifugation at 2000 rpm for 10 min at 4 °C, cytosolic and mitochondrial proteins were aspirated together with the supernatant, while the pellet (the nuclear fraction) was dissolved and incubated for 1 h in homogenization buffer containing Triton X 100 tenside in addition (Sigma-Aldrich-Merck KGaA, Darmstadt, Germany). A second centrifugation followed (14,000 rpm, 10 min, 4 °C), after which the supernatant contained the nuclear proteins which were then aspirated. The cytosol- and mitochondria-containing supernatant were centrifuged (10,000 rpm for 20 min at 4 °C), then the supernatant was collected, which contained the cytosolic fraction. From 10 μL of the supernatants (nuclear and cytoplasmatic), total protein concentration was measured with a spectrophotometer (FLUOstar Optima, BMG Labtech, Ortenberg Germany), using BCA assay (QuantiPro BCA Assay Kit, Sigma-Aldrich-Merk KGaA, Darmstadt, Germany). The remaining volume was mixed with Laemmli sample buffer (Sigma-Aldrich-Merck KGaA, Darmstadt, Germany) or was frozen to −80 °C. Proteins were separated according to their molecular weight using SDS-Polyacrylamide gel electrophoresis (12% gel, 25 mA for ~220 min). Thereafter, proteins were transferred (25 V, 90 min) to a nitrocellulose membrane (GE Healthcare, Darmstadt, Germany), blocked in 3% BSA solution (Sigma-Aldrich-Merck KGaA, Darmstadt, Germany), and incubated overnight with TBST solution containing primary anti-bodies (anti-HistoneH3 recombinant rabbit monoclonal antibody, detecting histone 3 (~17 kDa), Cat#ab1791 Abcam, Cambridge, UK; anti-beta-actin mouse monoclonal anti-body, detecting beta-actin (~42 kDa), Cat#A5316, Sigma-Aldrich-Merck KGaA, Darmstadt, Germany; anti-MMP9 rabbit polyclonal anti-body (~92 kDa), Cat#ab38898, Abcam, Cambridge, UK; anti-PARP1 polyclonal anti-body (~113 kDa), Cat#ab227244, Abcam, Cambridge, UK; GFAP rabbit polyclonal antibody (55, 48 kDa), Cat#ab7260, Abcam, Cambridge, UK; anti-NFκB p65 rabbit polyclonal anti-body (~64 kDa) Cat#ab16502, Abcam, Cambridge, UK). The following morning, membranes were washed with TBST (3 × 10 min), and secondary antibodies, conjugated with horseradish-peroxidase enzyme, were applied to the membranes (anti-mouse anti-body Cat#A4416; anti-rabbit anti-body Cat#A0545; both from Sigma-Aldrich-Merck KGaA, Darmstadt, Germany). To make the blots visible, WesternBright™ (Advansta Inc., Menlo Park, CA, USA), enhanced chemiluminescent substrate of the enzyme, and LiCor C-Digit^®^ blot scanner (LI-COR Inc., Lincoln, NE, USA), were used. Scanned images were evaluated with ImageJ software (version 1.51, National Institutes of Health, Bethesda, MD, USA), during which normalization to the background and standardization to a housekeeping protein (Histone H3 or beta-actin) were performed. Three Western blots of all treatment groups were analyzed (*n* = 4).

### 2.7. Histology: Hematoxylin–Eosin Staining

After the extermination of animals, the eye bulbs (of five animals per group) were immediately removed from the orbit, and the upper part of the eyeball was marked for later positioning. Then, the bulbs were injected with and immersed into 4 °C paraformaldehyde solution (PFA, pH 7.4, 4% in phosphate buffer: 10 g paraformaldehyde, 50 µL 10 N NaOH, 25 mL 10× PBS, 200 ml ddH_2_O) for 24 h to provide the appropriate fixation of the retina. On the next day, corneas were removed to guarantee the complete washout of the PFA, and tissue samples were washed for 1 hour in water. Thereafter, samples were stored in 70% alcohol until further histological processing. The next step was dehydration (70%, 90%, 100% ethanol), followed by clearing (xylene) and wax infiltration/embedding (Histowax, Histolab Products AB, Gothenburg, Sweden), and finally the paraffin-embedded eye tissue blocks were sectioned frontally with a microtome into 5 µm-thick sections. We further processed the sections which were localized near the optic disk. After deparaffinization and rehydration of the sections, hematoxylin–eosin (H & E) staining was performed as follows: the sections were stained for 10 min with hematoxylin (Gill-type, GHS2128, Sigma-Aldrich-Merck KGaA, Darmstadt, Germany). Sections were rinsed in running tap water for 10 min until they turned blue, and then they were stained with eosin for 5 min. Images were taken from the inferior part of the retina, near the optic disk, with Nikon Eclipse 80i microscope with a DS-Fi3 Microscope Camera attached, through a 40× objective (Nikon Plan Fluor 40×/0.75 DIC M/N2 ∞/0.17 WD 0.66). Measurements were made with the microscope software Nikon NIS-Elements BR (Ver5.41.00).

### 2.8. Statistical Analyses

For data analysis, GraphPad Prism software (version 8.0, GraphPad Software Inc., La Jolla, CA, USA) was used. First, Gaussian distribution was estimated with the Shapiro–Wilk normality test. Following this, according to the result of normality test, data were analyzed either with one-way analysis of variance (ANOVA) or non-parametric Kruskal–Wallis test. In the case of analyzing group values in different time points, a two-way analysis of variance was used. When the probability value was lower than 0.05, the comparisons were estimated as significant. The significance levels were indicated as follows: * *p* < 0.05; ** *p* < 0.01; *** *p* < 0.001; and **** *p* < 0.0001. All data are presented as mean ± standard error of the mean (SEM).

## 3. Results

### 3.1. Ocular Ultrasound

**Figure 1:**
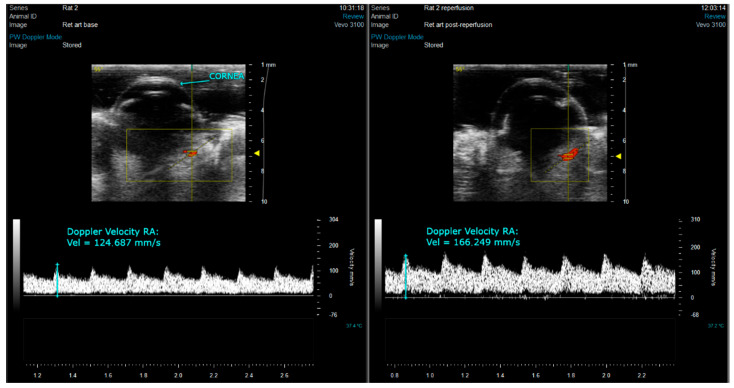
Representative ultrasound images showing blood supply of the eye bulb before and after ischemic ligation. Yellow box: Area of color doppler imaging. Vertical yellow line with short horizontal parallel yellow lines in the middle: gate of pulsed wave doppler (between the two short horizontal lines). Yellow dashed transverse line: angle of pulsed wave doppler. Red: Bloodflow towards the transducer. Blue line: measuring tool for maximal bloodflow velocity.

Retinal arterial blood flow evaluated by Color and PW Doppler echography was restored after the I/R injury. Peak blood flow velocities in the ciliary arteries varied between 100 and 300 mm/s, both at baseline and post-reperfusion phases, showing that reperfusion was complete after the injury (Figure 1).

### 3.2. Electroretinography

**Figure 2:**
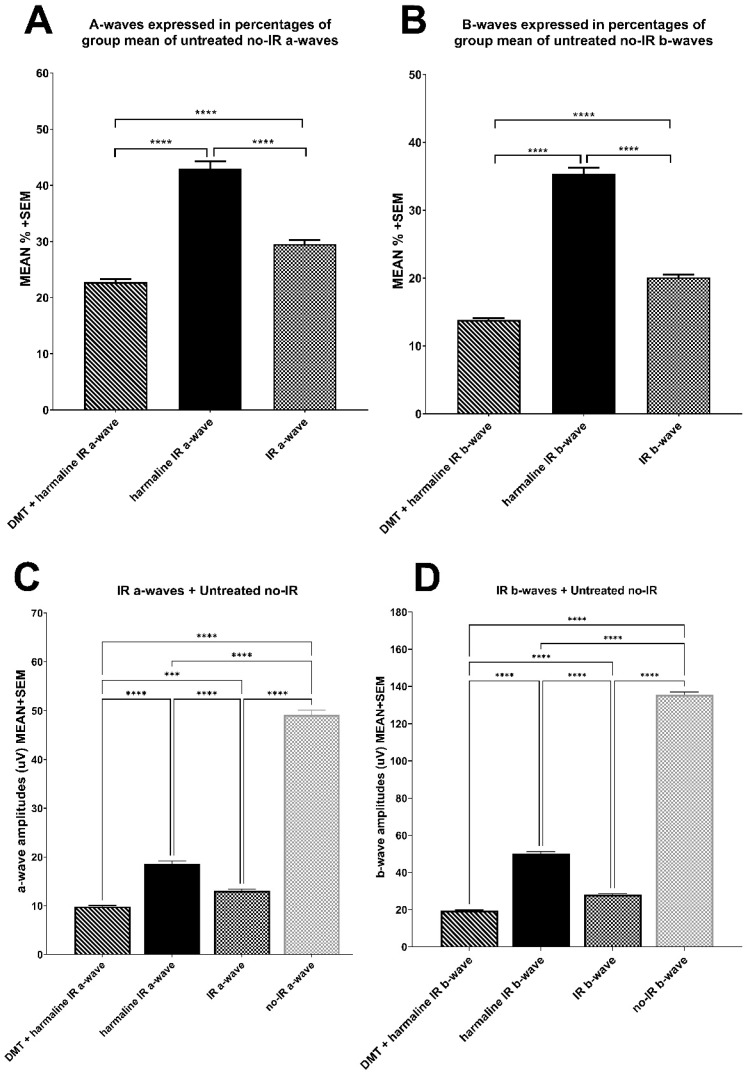
Electroretinography results. (**A**): a-waves expressed in percentages of group mean of untreated no-IR a-waves. (**B**): b-waves expressed in percentages of group mean of untreated no-IR b-waves. (**C**): absolute values of IR a-waves and untreated no-IR a-wave expressed in µV. (**D**): absolute values of IR b-waves and untreated no-IR b-wave expressed in µV. All results are plotted as mean + SEM. *** = *p* < 0.001; **** = *p* < 0.0001.

The highest values were observed in the group of harmaline-treated animals: here, a-waves were measured to be 42.92 ± 1.35, b-waves 35.38 ± 0.89 percentage of untreated, non-ischemic control values, both differing significantly (*p* < 0.0001) from ischemic–reperfused (IR) values, 29.49 ± 0.79 and 20.08 ± 0.45 for a- and b-waves, respectively. Combined treatment with DMT and harmaline together turned out to decrease a- and b-waves (22.76 ± 0.52 and 13.82 ± 0.24, both *p* < 0.0001) compared to non-ischemic values (see Figure 2 A,B). Figure 2 C,D show absolute values of a- and b-wave amplitudes in microvolts including untreated no-IR control.

### 3.3. Western Blot

**Figure 3:**
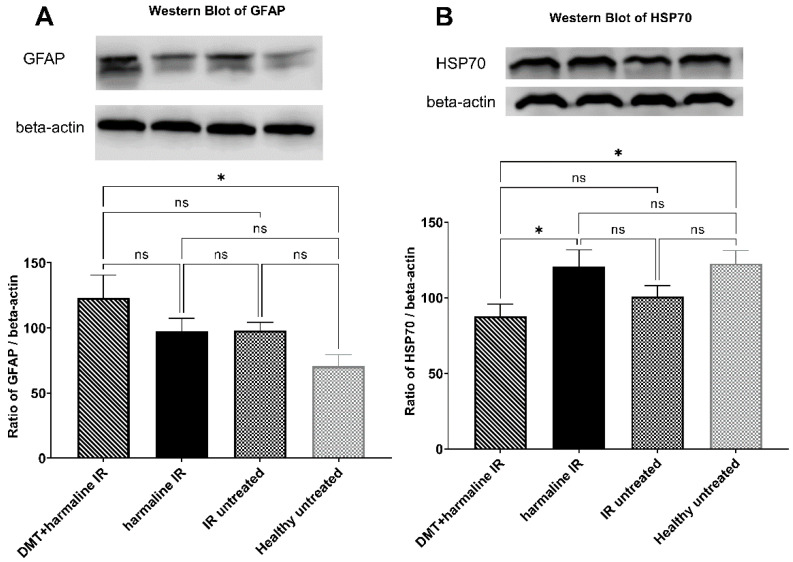
Western blot results. (**A**): Expression levels of Glial fibrillary acidic protein (GFAP) in the different groups. (**B**): Expression levels of heat shock protein 70 (HSP70) in the different groups. All results are plotted as mean percentages + SEM. ns = non-significant; * = *p* < 0.05.

According to the Western blot results, GFAP levels were significantly different between healthy untreated and DMT + harmaline-treated groups (70.59 ± 8.879 vs. 123.0 ± 17.56; *p* < 0.05). In contrast, there was no statistical difference between the healthy untreated and harmaline groups (70.59 ± 8.879 vs. 97.20 ± 10.16; *p* > 0.05). Although the I/R untreated group showed a seemingly higher level (97.93 ± 6.342) than the healthy untreated group, this difference did not reach the level of statistical significance either (Figure 3A). 

HSP70 protein also showed differential expression between the treatment groups. There proved to be a significant difference between healthy untreated and DMT + harmaline-treated groups as well as between Harmaline-treated and DMT + Harmaline-treated groups (122.6 ± 8.638 vs. 87.72 ± 7.993 *p* < 0.05 and 120.7 ± 10.92 vs. 87.72 ± 7.993 *p* < 0.05, for healthy untreated vs. DMT + harmaline-treated and harmaline-treated vs. DMT + harmaline-treated groups, respectively). Although the I/R untreated group showed a seemingly lower level (100.9 ± 7.191) than the healthy untreated group, this difference did not reach the level of statistical significance. There was no statistical difference between the healthy untreated and harmaline groups (*p* > 0.05) (Figure 3B).

**Figure 4:**
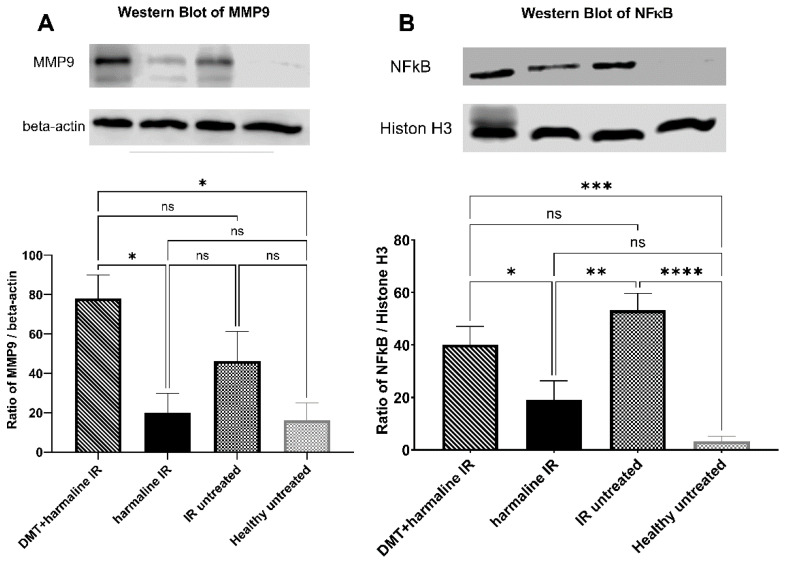
Western blot results. (**A**): Expression levels of Matrix metallopeptidase 9 (MMP9) in the different groups. (**B**): Expression levels of nuclear factor kappa-light-chain-enhancer of activated B cells (NFκB) p65 subunit in the different groups. All results are plotted as mean percentages + SEM. ns = non-significant; * = *p* < 0.05; ** = *p* < 0.01; *** = *p* < 0.001; **** = *p* < 0.0001.

Similar significances were observed in case of differences in MMP9 levels based on our Western blot method: significant differences were seen in comparisons of healthy untreated vs. DMT + harmaline-treated groups and harmaline-treated vs. DMT + harmaline-treated groups (16.11 ± 8.909 vs. 78.08 ± 11.85 *p* < 0.05 and 19.79 ± 9.925 vs. 78.08 ± 11.85 *p* < 0.05, for healthy untreated vs. DMT + harmaline-treated, and harmaline-treated vs. DMT + harmaline-treated groups, respectively). In this case, however, levels of MMP9 protein changed the opposite way as that of HSP70: protein levels were decreased in healthy untreated and harmaline-treated groups and increased in I/R untreated and DMT + harmaline-treated groups. Although there was no significant difference between the healthy untreated and harmaline groups, the I/R untreated group also did not differ significantly from either of these groups (16.11 ± 8.909, 19.79 ± 9.925, and 46.30 ± 15.06 for healthy untreated, harmaline-treated, and I/R untreated groups, respectively; *p* > 0.05 in each comparison) (Figure 4A).

In the case of NFκB p65 protein, the IR untreated group differed significantly from both the healthy untreated and the harmaline-treated I/R groups (53.21 ± 6.409 vs. 3.350 ± 1.850, *p* < 0.0001 and 53.21 ± 6.409 vs. 19.19 ± 7.140, *p* < 0.01, for I/R untreated vs. healthy untreated and I/R untreated vs. harmaline-treated groups, respectively). NFκB p65 levels of the DMT + harmaline-treated group were significantly higher than levels of both harmaline-treated and healthy untreated groups (40.19 ± 6.864 vs. 19.19 ± 7.140, *p* < 0.05 and 40.19 ± 6.864 vs. 3.350 ± 1.850, *p* < 0.001 for DMT + harmaline-treated vs. harmaline-treated groups and DMT + harmaline-treated vs. healthy untreated groups, respectively). There was no statistical difference between healthy untreated and harmaline-treated groups (3.350 ± 1.850 vs. 19.19 ± 7.140) (Figure 4B).

**Figure 5:**
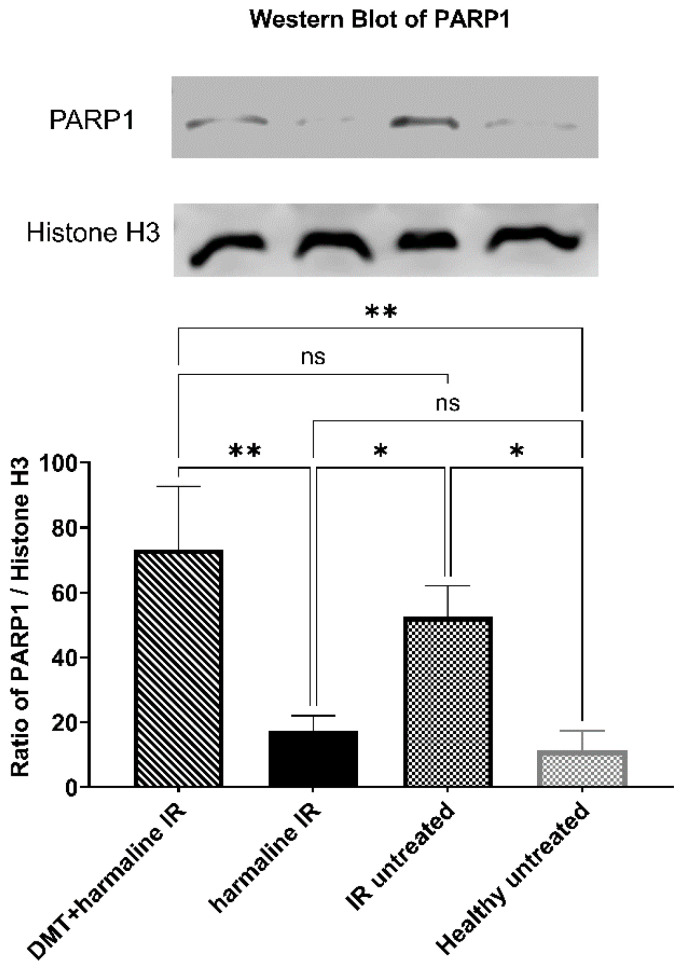
Western blot results: Expression levels of Poly [ADP-ribose] polymerase 1 (PARP1) in the different groups. All results are plotted as mean percentages + SEM. ns = non-significant; * = *p* < 0.05; ** = *p* < 0.01.

Similarly, poly-ADP-ribose polymerase (PARP) 1 protein levels of I/R untreated and DMT + harmaline-treated groups were higher (52.47 ± 9.597 and 73.26 ± 19.42, respectively), while that of healthy untreated and harmaline-treated groups were lower (11.22 ± 6.145 and 17.41 ± 4.640). There were no statistically significant differences between the group-pairs above (*p* > 0.05 in both I/R untreated vs. DMT + harmaline and healthy untreated vs. harmaline-treated comparisons). In contrast to this, differences proved to be significant in the case of healthy untreated vs. I/R untreated (*p* < 0.05), I/R untreated vs. harmaline (*p* < 0.05), harmaline vs. DMT + harmaline (<0.01), and DMT + harmaline-treated vs. healthy untreated (*p* < 0.01) comparisons (Figure 5.).

### 3.4. Histology

**Figure 6:**
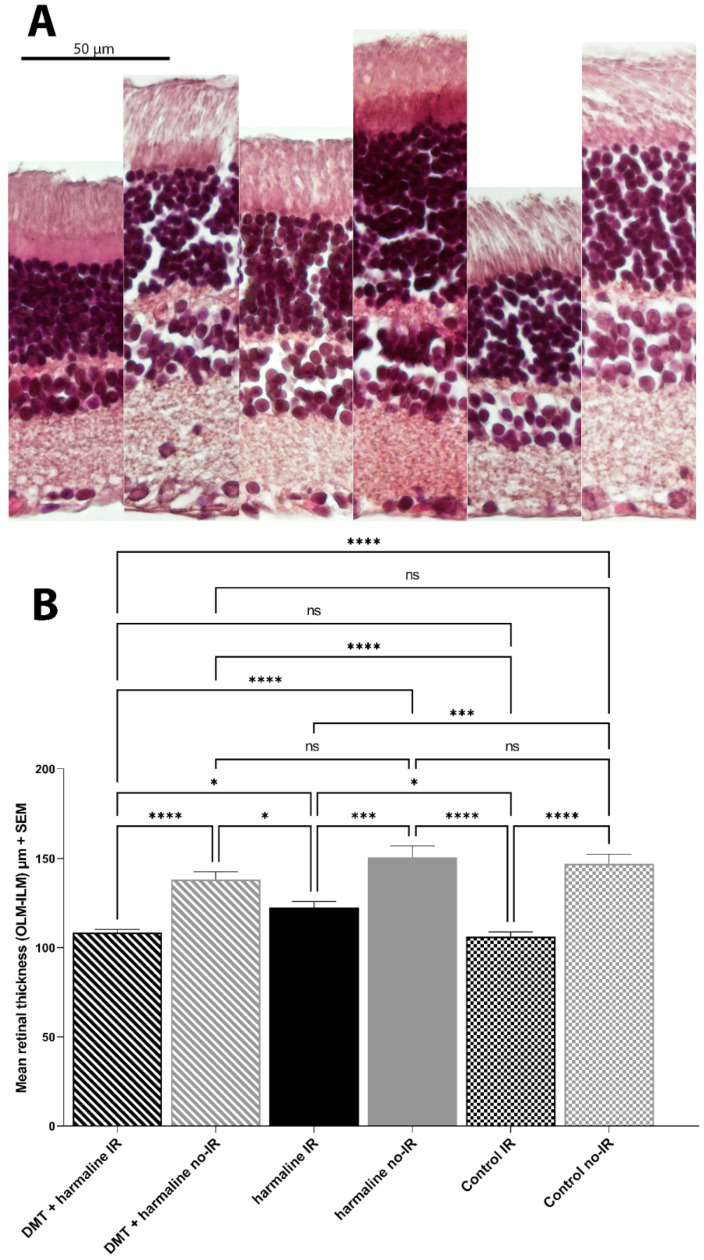
Retinal thickness in the different treatment groups. (**A**): Representative images of retinal thickness in the different treatment groups as seen under the microscope during histological analysis. (**B**): Graph of group mean retinal thickness as measured in sections of the different treatment groups during histological analysis. The order of the sections and the graphs are identical. All results are plotted as mean percentages + SEM. ns = non-significant; * = *p* < 0.05; *** = *p* < 0.001; **** = *p* < 0.0001.

Based on the histology results (Figure 6), there were significant differences between the thickness of the whole retina in the different groups. In the case of the control group, thickness of the ischemic–reperfused retina proved to be significantly thinner compared to its respective non-ischemic value (106.2 ± 2.608 µm vs. 147.0 ± 5.394 µm; *p* < 0.0001). The same significant difference could be observed in the two treated groups: ischemic retinal thickness was decreased compared to the non-ischemic values (108.5 ± 1.716 µm vs. 138.2 ± 4.382 µm and 122.5 ± 3.267 µm vs. 150.6 ± 6.283 µm for ischemic vs. non-ischemic values of DMT + harmaline- and harmaline-only-treated groups, respectively; *p* < 0.0001 in both comparisons). If we compare the non-ischemic values of the different treatment groups, no differences were observed between them (138.2 ± 4.382 vs. 150.6 ± 6.283 vs. 147.0 ± 5.394 µm for DMT + harmaline-treated, harmaline-only-treated, and control groups, respectively; none of the values are significantly different from the others in the comparisons). In comparing ischemic retinal thickness values, significant differences exist: the harmaline-treated group proved to be significantly thicker than both the control group and the DMT + harmaline-treated group as well (122.5 ± 3.267 vs. 106.2 ± 2.608 and 108.5 ± 1.716 µm for harmaline vs. control and DMT + harmaline-treated groups, respectively; *p* < 0.05 in both comparisons). There were no significant differences between the treatment and control group I/R values of DMT + harmaline-treated and control groups (106.2 ± 2.608 vs. 108.5 ± 1.716 µm for DMT + harmaline-treated group vs. control; *p* > 0.05).

## 4. Discussion

Several eye diseases are known to diminish vision by either decreasing optical transparency or damaging neural integrity [21]. Ischemia/reperfusion injury of the eye provokes a cascade of deleterious events that include energy failure, excitotoxic damage, calcium imbalance, oxidative stress, inflammatory immune response, and eventually cell death. The consequential activation of the immune system and stress response is crucial to repair and maintain cellular homeostasis, but the associated inflammation and the arising oxidative stress during I/R injury can lead to significant tissue destruction [22]: the thickness of the whole retina decreases after an ischemic attack, indicating a severe degradation of all retinal layers [23].

The ischemic eye diseases are a major cause of vision impairment; however, there is no currently available, effective treatment for I/R-based eye disorders [2]. N, N-Dimethyltryptamine, due to its Sigma-1 receptor agonist activity, is considered to be a promising drug candidate in the treatment of I/R-related diseases [8,10,11,24]. DMT is an indole alkaloid that naturally occurs in several plants (e.g., Psychotria viridis and Diplopterys cabrerana) and is used for religious and healing rituals or recreational purposes by humans as a brew (Ayahuasca) due to its psychotropic effects when ingested [24]. Due to its effect on a subset of serotonin (5-HT) receptors, it has been suggested to be effective in mood disorders, e.g., depression [25]. Besides, it also acts on Sig-1R, which is considered a "pluripotent modulator" that controls cell survival and differentiation and also, in case of endoplasmic reticulum stress, initiates synthesis of anti-stress and antioxidant proteins in the nucleus [7]. Accordingly, activation of Sig-1R reduces damage caused by hypoxia and oxidative stress. For this reason, DMT was studied by many authors in hypoxia. Szabo. A. et al. demonstrated that DMT exerts its protective effect against hypoxia in human iPSC-derived cortical neurons and microglia-like cells in an HIF-1α-independent manner by regulation of Ca2+ signaling [26]. Its protective effect against ischemia/reperfusion injury has been studied in kidneys, where it mitigated metabolic changes during and after I/R [10]. Its neuroprotective properties were proven in several studies: due to its Sig-1R agonist activity, DMT could attenuate several pathophysiological aspects of ischemic injury in the rat brain [11,24]. Furthermore, Sig-1Rs are localized on diverse cell types of the retina and play a protective role against stress-induced cell loss [27]. Based on these studies we wanted to test DMT on retinal ischemia/reperfusion injury as well.

Harmaline is a β-carboline that works as a reversible inhibitor of MAO-A and is one of the cardinal components of Ayahuasca brew, besides Harmine and tetrahydro-harmine. β-carbolines can potentiate the neurobehavioral effect of DMT, although their overall physiological effect is more complex because they may be neurologically active too: Harmala alkaloids exert psychotropic properties as well [7]. Although Harmine is more lipophilic and can reach a higher concentration in the brain, harmaline elimination from the central nervous system is slower and it has a longer half-life than Harmine [28]. Harmaline gained attention in the last decade and has been reported to exert multiple pharmacological effects, including antimicrobial, antiplatelet, antitumoral, hypothermic, and vasorelaxant properties [12]. According to a recent study, harmaline and Harmine could effectively improve cognitive function in scopolamine-treated mice: besides their MAO-A-inhibitory effect, they inhibit acetylcholinesterase, induce choline acetyltransferase activities, and exert anti-inflammatory properties [17].

MAOs are FAD-dependent enzymes localized at the outer mitochondrial membrane, responsible for the oxidative breakdown of key neurotransmitters. During the oxidative deamination of monoamines by MAOs, corresponding aldehydes and hydrogen peroxide generate oxidative stress, which is potentially a risk factor for neuronal loss in neurodegenerative disorders. Furthermore, MAO-A enzymes may have a role in apoptosis [29] and may down-regulate basal mitochondrial function as well [30]. According to a recent study, MAO-A is upregulated in glaucoma, strengthening the idea that the MAO-A enzyme has a crucial role in degenerative eye diseases [31].

Thus, based on several scientific articles, we hypothesized that DMT might have retinoprotective effects and we applied harmaline to maintain therapeutic DMT concentration. To be able to distinguish any possible beneficial effect of harmaline from the effects of DMT, we also created a harmaline-only-treated group. At the beginning of the experiment, we also assumed a possible synergistic retinoprotective effect of DMT and harmaline, the two components of Ayahuasca brew, in retinal ischemia/reperfusion injury.

However, interestingly, only harmaline treatment could mitigate significantly the retinal layer-thickness reduction after I/R, and only this alkaloid could preserve the retinal function to some extent. Based on our ERG results, harmaline maintained higher level of retinal function after I/R while DMT and harmaline co-administration even decreased the amplitudes of ‘a’ and ‘b’ waves compared to the untreated ischemic group, which may indicate potential detrimental effects on the eye. To find an explanation for this paradox—accepting the fact that in the serotonergic pathway DMT and harmaline work synergistically (to increase serotonergic effects)—we tried to find new targets on which these two agents act inversely, or at least differently; thus, we investigated the expression of several proteins in the eye which are important in cell death (PARP1), inflammation (NFκB), tissue destruction (MMP9), tissue remodeling (GFAP), and maintaining cellular homeostasis (HSP70).

PARPs are crucial regulators of cellular homeostasis and they modify several cellular processes including apoptosis, pro-inflammatory responses, mitochondrial function, and metabolic stress, which are also involved in I/R injury. Poly-ADP-ribose polymerase 1 is the most intensively studied member of the PARP superfamily [32] and has a vital role in DNA repair as its hyperactivation can cause a particular type of programmed cell death, which—according to several studies—may have a significant role in retinal damage, inherited retinal diseases, age-related macular degeneration, and ischemia/reperfusion of the eye [33]. In our study, HT decreased PARP1 expression in the eye bulb compared to the VT and DHT groups, which can contribute to the retinoprotective effect of harmaline. Similarly, several experiments have proven that the PARP1 inhibition by other agents decreases inflammatory response in microglia, indicating that PARP1 is involved in microglial activation [34], while gliosis might be an early and sensitive marker of I/R injury of the eye [35].

Furthermore, PARP1 enhances the activation of CNS immune cells and influences the expression of several transcription factors, including NF-κB, p53, and AP-1, which are involved in inflammatory response and tissue regeneration [36]. NFκB is defined as a family of five members/subunits playing a critical role in the regulation of multiple cellular processes including inflammation, tissue regeneration, and cell cycle [37]. The most abundant form of NFκB is modulated via the canonical pathway and is comprised of the heterodimer of p65 and p50 subunits. This heterodimer is a major regulator of inflammatory processes [38], and its activation in microglia has prime importance in the production of ROS and pro-inflammatory cytokines (including IL-1β, interferon-γ, and TNF-α) that can cause neuronal damage [39]; accordingly, the inhibition or downregulation of NFκB has a retinoprotective effect [40,41]. As we expected, I/R injury increased the level of native NFκB p65 subunit in the eye. Harmaline was able to prevent the excessive expression of p65, which can potentially alleviate inflammatory response in I/R injury and preserve the retinal function seen in ERG results of HT groups. Surprisingly, in the DHT group, the NFκB protein level was not significantly decreased compared to VT group, which indicates that DMT can counterbalance the anti-inflammatory potential of harmaline. A reason for this might be the high PARP1 concentration of DHT group compared to HT or even to VT groups, although the latter comparison did not show statistically significant difference.

Besides its NFκB activator effect, PARP1 can contribute to blood–retina barrier disruption with the stimulation of Matrix metalloproteinases (MMPs). The expression of MMP-9 can be enhanced by the binding of both NFκB and AP-1, a coactivator of both of which is PARP1 [42]. MMPs are responsible for extracellular matrix degradation and tissue remodeling [43]. In the case of transient intraocular pressure elevation, which leads to retinal ischemia, the MMPs are highly expressed in the trabecular meshwork, aqueous humor, retina, and optic nerve: there is evidence that MMP9 overexpression contributes to retinal damage in I/R injury [44]. Consequently, the inhibition of MMPs could be a promising therapeutic approach in the treatment of ischemic retinal diseases. In our experiment, HT could alleviate the MMP9 overexpression, since there was no difference between HC and HT groups. Opposed to this, the MMP9 expression in the DHT group was significantly higher than in the HT and HC groups, which further corroborates NFκB and PARP1 results. 

Astrocytes play a crucial role in the overall reaction of the retina to injury and disease. I/R injury of the eye activates astrocytes to secrete MMPs that may participate in matrix remodeling and degradation of the retina [43]. GFAP is a widely used indicator of astrocyte activation due to its substantial increase in expression observed in most pathological conditions, including neurodegeneration and neuronal damage [45]. According to a recent study, in retinal damage, preservation of photoreceptors was observed when GFAP was inhibited [46]. In our investigation, the GFAP expression did not change significantly in the HT group compared to the VT or even to HC animals, but DMT and harmaline together probably enhanced the astrocyte activity, further indicated by the significant GFAP overexpression in the DHT group compared to HC. 

Heat shock protein 70s (Hsp70s) are ubiquitous chaperones responsible for the regulation of activity, folding, and disassembly of protein complexes. Under pathological conditions, Hsp70s protect the cell from the harmful effects of a wide range of proteotoxic noxa [47]. Hsp70 induction has several therapeutic benefits: they may exert neuroprotective properties inhibiting pro-inflammatory cytokine release and astrogliosis [48]. Hsp70 exerts anti-inflammatory properties, since it has been shown to inhibit several proinflammatory factors such as NF-kB, MMPs, and ROS, resulting in an anti-inflammatory state. Overexpression of Hsp70 has been shown to decrease NF-kB activation in astrocytes [49]. According to our Western blot results, DMT counterbalanced the Hsp70 expression-increasing effect of harmaline in the eye: HSP70 expression was significantly lower in the DHT group compared to HT group, which showed a similar level to the HC group, and there was no significant difference between HT and HC. Our electroretinographical measurements correspond with the results of Western blot analysis. In the HT group, the amplitudes of the ‘a’ and ‘b’ waves were higher than in the VT and DHT groups, indicating the protective effect of harmaline. Molecular background of this phenomenon may be the increased expression of the protective chaperone Hsp70 and decreased expression of PARP1, NFκB, and MMP9, which are crucial participants in the apoptosis pathway, inflammatory response, and tissue destruction. However, DMT counterbalanced this protective effect. 

The role of 5-HT is debated in retinal diseases. Systemic MAO-A enzyme inhibition leads to reduced degradation of 5-HT and norepinephrine [14]. The molecule structure of DMT shows near resemblance to 5-HT, and consequently it acts mainly on 5-HT_1A_, 5-HT_2A_, and 5-HT_2C_ receptors [4,6]. The co-administration of MAO-A inhibitor, harmaline, and DMT may lead to excessive stimulation of 5-HT receptors. 

Agonists of 5-HT_1A_ receptor are considered neuroprotective agents. Agonism of 5-HT_1A_ is neuroprotective in animal models of CNS ischemia, traumatic brain injury, excitotoxicity, Parkinson’s disease, delayed progression of motor neuron degeneration, and reduced lipid peroxidation in a rat epilepsy model. Its beneficial properties have also been proven in light-induced retinal damage [50]. The mechanisms underlying the protective effects of 5-HT_1A_ agonism in the CNS and retina have not been entirely explored: it might enhance the expression of antioxidant enzymes, antiapoptotic proteins, and inhibitors of apoptosis proteins [50]. However, on the other hand, 5-HT receptor activation may initiate a deleterious cascade. Serotonin overproduction may contribute to valvular heart disease, coronary artery disease, peripheral vascular disease, and diabetic nephropathy [51]. A recent study revealed that the expression of the 5-HT_2A_ receptor and MAO-A in renal I/R injury increases and the 5-HT level is also elevated. These findings indicate that 5-HT and its degradation system are also important in I/R damage. Furthermore, the inhibition of 5-HT_2A_ led to decreased MAO-A expression and mitigated the oxidative damage [52]. The increase in ROS content in I/R-damaged tissue is probably due to the degradation of 5-HT by MAO-A, which can be inhibited by a 5-HT_2A_ receptor antagonist, 5-HT synthase inhibitors, or an MAO-A inhibitor [14,52]. In BALB/c mice, intraperitoneal application of 5-HT_2A_ receptor antagonist preserved the retinal thickness and integrity in bright light (10,000 lux) Exposure (LE)-mediated retinal damage [53]. The 5-HT_2C_ receptor (5-HT_2C_R) activation may also contribute to I/R injury: in isolated retinal ganglion cells, the 5-HT_2C_R blockade was protective against glutamate-induced cell death [54].

The deleterious effect of DMT was entirely unexpected. Its potential neuroprotective properties—such as effects on Sig-1R and 5-HT_1A_R, seen in other organs—probably could not predominate in the eye. The eye is an acutely sensitive sensory organ [21]. I/R injury activates inflammatory response, causes tissue destruction, and provokes apoptosis in the eye, especially in the retina [22]. Based on our findings, MAO-A enzyme inhibitor harmaline could alleviate the hazardous effect of I/R injury. Sticking to the theory of acting through the serotonergic pathway, we hypothesize that the MAO-A inhibition might reduce the oxidative stress after ischemia with the inhibition of oxidative deamination (5-HT degradation system). Considering that there are controversial data in the scientific literature about the effect of 5-HT in pathological conditions, we suppose that there might exist a delicate balance between normal and excessive function of 5-HT in the eye, which balance was upset by DMT and harmaline together. In the HT group, the increased 5-HT concentration might not reach a deteriorative concentration; indeed its increase turned out to be even beneficial, while in the DHT group the same high dose of 5-HT together with the 5-HT receptor agonist DMT could cause receptor over-activation. There is increasing evidence that selective serotonin reuptake inhibitors (SSRI) cause decreased visual acuity due to retinal pigment epithelium (RPE) atrophy and reduction in the foveal and the perifoveal macular ganglion cell complex thickness [54]. Furthermore, DMT has a high affinity to 5-HT_2A_ receptors, which are localized in high number in RPE and found in high density in the claustrum, a region connected to the visual cortex [55]. The unfavorable effect of DMT on the visual organ may be mediated by 5-HT_2A_ (over)activation. In addition, DMT can inhibit serotonin transport (SERT), further increasing 5-HT concentration in the retina [56].

Another hypothesis can be, still staying with the theory of both treatments acting through the serotonergic pathway, that DMT may act differently in the eye—or even in the opposite manner—compared to other organs, either due to a different subset of (e.g.,) 5-HT receptors in the eye, keeping in mind that several 5-HT receptor subtypes exist, or due to a different (“retinal”) receptor subunit-composition—also known as isoform or variant—of a specific receptor DMT acts on. Benzodiazepines are good examples of the latter: by binding to different isoforms of GABA_A_ receptors they exert different effects (anxiolytic, anticonvulsant, hypnotic, etc.) in different brain regions [57,58]. Furthermore, articles exist about DMT activating Sig-1R, and other articles demonstrate that Sig-1R can be found in the eye; however, whether DMT acts on retinal Sig-1 receptors has not been investigated yet. It has been suggested that more subtypes of Sig-R may exist, and with different sensitivity: a low ligand dose may activate one, higher concentration another [59,60,61]. DMT was proven to act on Sigma-2 (Sig-2R) receptors as well [62]. However, Sig-2R activation may activate cell death [63] and may even have a key role in neurodegenerative diseases as well [64]. Clear distribution and function of Sigma-2 receptor in the eye has not been thoroughly investigated yet.

However, if we accept the facts that 5-HT receptors (most likely) transmit anti-ischemic effect (in the eye as well) and both DMT and harmaline increase serotoninergic signal transduction synergistically, then we derive our third hypothesis for the presented, unexpected results of co-treatment. We have to consider the possibility of an unknown receptor-mediated or allosteric effect, or even a receptor-independent signal-transductional cascade of DMT in the mammalian eye. This hypothesis is supported by our results. Based on our experiments, DMT and harmaline exert opposite effects on ocular proteins such as PARP1, NFκB, MMP9, or HSP70, each having a severe role in different mechanisms of eye-ischemia-related pathologies: cell death, inflammation, tissue destruction, and oxidative stress.

DMT is a promising drug candidate in treating several diseases, including PTSD, depression, stroke, and renal I/R injury [11,24,65]. However, our observations necessitate further investigations with it, especially in the eye. On the other hand, considering the positive results of harmaline treatment, exploration of therapeutic potential of this MAO inhibitor in I/R-related eye diseases is advised.

## 5. Conclusions

Based on the existing scientific literature, DMT was supposed to act beneficially in eye I/R. To inhibit its rapid breakdown by MAO-A enzyme, we co-administered DMT with harmaline, as used in Ayahuasca brew. However, our present experiment took an unexpected turn. Although harmaline alone exhibited retinoprotective effect in I/R injury of the eye—according to our electroretinographical measurements (ERG) and histological analysis—co-administration of DMT counterbalanced this beneficial effect. Whether such effects of DMT are related to a sensitive, fragile 5-HT homeostasis in the eye, 5-HT receptor overstimulation, or 5-HT_2A_ activation, is to be investigated: a hypothesis is that DMT might cause the overactivation of the 5-HT system, shifting the reparative process into an unfavorable direction. An even more likely hypothesis is, however, that the demonstrated effects are unrelated to 5-HT, as both DMT and harmaline act synergistically to increase serotonergic effects, while in our results they proved to exert opposed effects, which were further supported by Western blot analyses. Here, we demonstrated that DMT and harmaline exert opposite effects on expression of different important ocular proteins—PARP1, NFκB p65, MMP9, or HSP70, each having a critical role in different mechanism of eye-ischemia-related pathologies: cell death, inflammation, tissue destruction, and oxidative stress. Taking into consideration the positive results of harmaline treatment, we suppose that the use of this MAO-A-inhibitor agent might provide a potential therapeutic strategy in the prevention and treatment of I/R-related eye disorders.

## Figures and Tables

**Table 1:** Experimental structure.

Groups	Number of Animals	Treatment	Examinations
1.	10 male SD rats	DMT (sc.) + harmaline (po.) (7 days) = DHT	Electroretinography + Histology + Western blot
2.	10 male SD rats	harmaline (po.) (7 days) = HT	
3.	10 male SD rats	vehicle-treated group = VT (left ligated eyes = untreated control; right non-ischemic eyes = healthy control)	

## Data Availability

The data presented in this study are available on request from the corresponding author.
